# A case of cerebral hyperperfusion following spontaneous recanalization of occluded middle cerebral artery: Reperfusion injury or true cerebral hyperperfusion syndrome?

**DOI:** 10.1097/MD.0000000000006740

**Published:** 2017-04-28

**Authors:** Ziqi Xu, Jiajia Zhou, Hui Liang, Benyan Luo, Ping Liu

**Affiliations:** Department of Neurology, The First Affiliated Hospital, College of Medicine, Zhejiang University, Hangzhou, China.

**Keywords:** cerebral hyperperfusion, ischemic stroke, recanalization case report, reperfusion injury

## Abstract

**Background:**

Cerebral hyperperfusion syndrome (CHS) and reperfusion injury are distinct pathological phenomena.

**Case summary:**

We present the case of a young ischemic stroke patient with middle cerebral artery (MCA) occlusion and spontaneous recanalization. Follow-up transcranial Doppler ultrasound showed high velocity flow in the left MCA, and neuroimaging revealed infarction, brain edema, artery dilatation, and hyperperfusion, consistent with both CHS and reperfusion injury.

**Conclusion:**

In cases with signs of both CHS and reperfusion injury, we speculate that CHS may be both a contributor to and a manifestation of reperfusion injury.

## 1 Introduction

Cerebral hyperperfusion syndrome (CHS) and reperfusion injury are separate clinical entities with distinct pathophysiological features. CHS may be observed following carotid endarterectomy and carotid artery stent, and is associated with significant morbidity and mortality. Imaging presentation includes brain edema and hemorrhage, whereas transcranial Doppler (TCD) ultrasound shows higher velocity than in the contralateral vessel.^[1–3]^ Alternatively, reperfusion injury is not well studied during the acute stage following artery recanalization, but is also associated with hemorrhagic transformation (HT) and brain edema.^[4]^ The 2 phenomena have distinct etiologies and are differentiated easily although rarely in clinical practice. It is still uncertain if CHS and reperfusion injury can occur simultaneously. Here, we present the case of a young ischemic stroke patient with left MCA occlusion presenting with signs of both CHS and reperfusion injury at follow-up.

## 2 Case presentation

This study was a prospective and observational study, approved by ethics committee of the First Affiliated Hospital of Zhejiang University and all methods were performed in accordance with the relevant guidelines and regulations.

**Figure 1:**
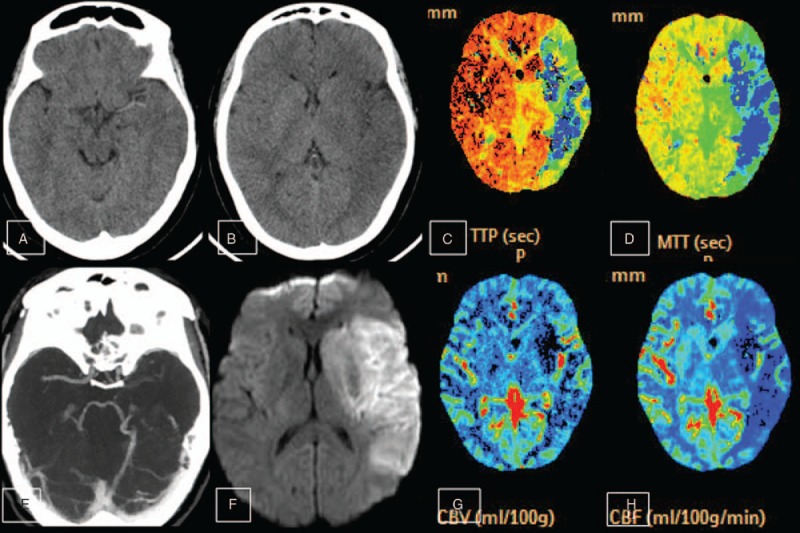
Emergency cranial multiple mode CT and diffusion-weighted images on hospital presentation (day 1). (A, B) Cranial CT showing left middle cerebral artery (MCA) hyperdensity sign with low brain tissue density at admission. (C, D, G, H) CT perfusion showing low perfusion throughout the whole left MCA distribution. (D) Cranial CT angiography (CTA) showing left MCA occlusion. (F) Diffusion-weighted imaging (DWI) showing high signal throughout the MCA distribution, indicating acute cerebral infarction. CT = computed tomography, CTA = CT angiography, DWI = diffusion-weighted imaging, MCA = middle cerebral artery.

A 28-year-old female was transferred to our Emergency Department with aphasia and right limb weakness of about 6 hours’ duration. The patient had no medical history of hypertension, diabetes mellitus, heart disease, or blood disease. National Institute of Health stroke scale (NIHSS) score was 22. Emergency multiple-mode cranial computed tomography (CT) showed left MCA hyperdensity sign (Fig. 1A) and low brain tissue density over more than 1/3 of the MCA territory (Fig. 1B). CT angiography revealed left MCA occlusion (Fig. 1E), whereas CT perfusion imaging showed cerebral hypoperfusion over the entire left MCA territory (Fig. 1C, D, G, and H). Due to unknown stroke onset time and the large region of low brain tissue density on cranial CT, the patient did not receive intravenous thrombolysis and endovascular therapy. Diffusion-weighted magnetic resonance imaging (DW-MRI) confirmed acute ischemic stroke (Fig. 1F). The patient was transferred to the Department of Neurology Ward Stroke Unit for further treatment.

**Figure 2:**
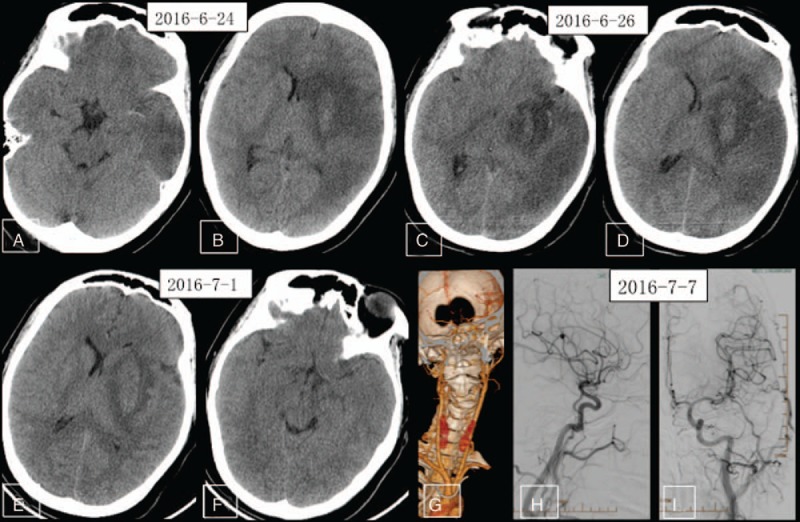
Follow-up cranial CT, CT angiography (CTA), and digital subtraction angiography (DSA). (A, F) Repeated cranial CT showing large infarction lesion with brain edema and HT; (G) repeated CT angiography showing spontaneous recanalization of the occluded left MCA complicated by MCA dilatation (but normal cervical artery structure). (H, I) Digital subtraction angiography (DSA) showing spontaneous recanalization of the left MCA with artery dilatation. CT = computed tomography, CTA = CT angiography, DSA = digital subtraction angiography, DWI = diffusion-weighted imaging, HT = hemorrhagic transformation, MCA = middle cerebral artery.

**Figure 3:**
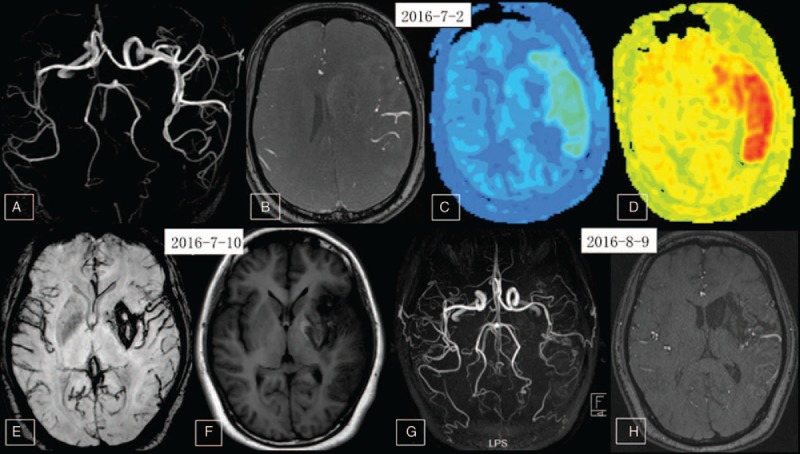
Follow-up MR angiography (MRA), arterial spin labeling (ASL), and susceptibility weighted imaging (SWI) results. (A) Time of flow MRA (TOF-MRA) showing marked left MCA dilatation at 9 days after disease onset; (B) TOF-MRA source image also indicated left MCA dilatation; (C, D) ASL perfusion image showed left MCA distribution hyperperfusion; (E, F) repeated MR image at about 2 weeks post-onset (C: SWI, F: T1-weighted image) showing large infarction of the left MCA distribution with HT and brain edema. Results show left infarction lesion with reduced HT and brain edema; (G,F) Repeated MRA and source image showing that the dilated left MCA recovered to normal but artery branch number was lower than the right MCA.ASL = arterial spin labeling, HT = hemorrhagic transformation, MCA = middle cerebral artery, MRA = MR angiography, TOF-MRA = time of flow MRA.

Neurologic examination showed somnolence, dysphonia and dysphagia, sensitive bilateral pupil light reflex, eyes right gaze disorder, shallow right nasolabial fold, grade 0 right upper and lower limb muscle strength, normal left upper and lower limb muscle strength, positive right Babinski sign, and normal left Babinski sign. The patient was unable to cooperate for sensory examination. The NIHSS score was confirmed as 22. The patient received conventional medical treatment including mannitol, albumin, and furosemide for alleviating brain edema. A nutrient solution was administered through a gastric tube. Ganglioside was infused to protect against further brain injury and rosuvastatin to protect the vascular endothelium. A second-day repeated cranial CT showed MCA distribution infarction with brain edema and hemorrhagic transformation (Fig. 2A and B). Through strict medical management, the patient's medical condition improved. Third- and tenth-day repeated cranial CT also found a large infarct core complicated with brain edema and HT (Fig. 2C, F), whereas CT angiography indicated normal cervical artery patency (Fig. 2G). Laboratory tests and further imaging exams were conducted to elucidate the cause of ischemic stroke. Laboratory test results are detailed in the Table 1. Echocardiography was normal and first-time Holter electrocardiography revealed sinus rhythm of the heart with occasional ventricular premature beats, paroxysmal ventricular tachycardia, and frequent atrial premature beats with paroxysmal atrial tachycardia and intermittent T wave change. A TCD saline bubble study was negative and also indicated that peak systolic velocity (PSV) of the left MCA was greater than the right MCA (257 cm/s vs 135 cm/s). Cerebral digital subtraction angiography was also conducted to assess etiology, but results showed dilated left MCA with no vascular malformations (Fig. 2H and I). In light of medical history, laboratory examination results, infarct pattern, and cerebral angiography, the patient was diagnosed with probable cerebral embolism of unknown origin. Due to the large infarct lesion and HT, the patient did not receive antithrombotic therapy. A 1-week MR angiography study showed spontaneous complete recanalization of the left MCA and MCA dilatation (Fig. 3A and B). Arterial Spin Labeling (ASL) images showed higher blood flow of the left MCA distribution than the right (Fig. 3C and D). A repeated Holter examination about 2 weeks after presentation showed sinus rhythm of the heart with occasional ventricular premature beats and atrial premature beats. A 2-week repeated TCD revealed that the velocity of the left MCA had improved to 137 cm/s, about equivalent to the right MCA (141 cm/s). Two-week repeated susceptibility weighted imaging (SWI) showed hemosiderosis associated with HT, and MR images showed alleviated brain infarct core and brain edema (Fig. 3E and F). The patient's general condition was also improved, with NIHSS score falling to 14. The patient was then transferred to a rehabilitation center. Six weeks later, repeated MR angiography showed similar bilateral MCA structure but fewer branches from the left MCA (Fig. 3G and H).

**Table 1:**
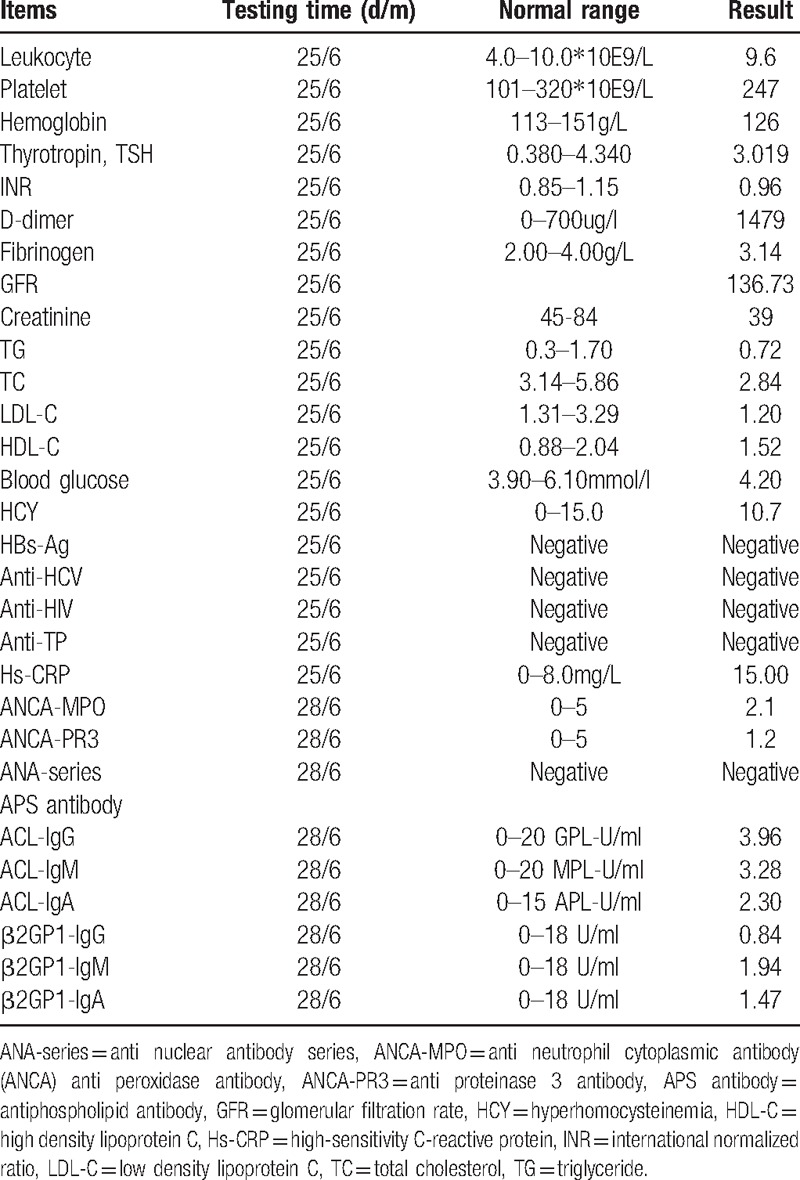
Laboratory test result of the patient.

## 3 Discussion

Stroke incidence has increased in young adults due to the rise in obesity and other lifestyle-related risk factors. In 1 series, the prevalence of large vessel atherosclerosis was about 19.8% in ischemic stroke patients aged 45 to 54 years.^[5]^ Atherosclerotic disease and cardioembolism are common reasons for early stroke.^[6]^ In our patient, however, cerebral vasculitis, blood diseases, rheumatic diseases, and acquired thrombophilia were ruled out by laboratory tests. Normal echocardiography and TCD saline bubble study results also helped rule out cardiac structural diseases such as patent foramen ovale. Combined with the results of DCG and the characteristics of recanalization on angiography, the patient was finally diagnosed with probable cerebral embolism of unknown origin. It is regrettable that we had no ability to test for blood protein C and S.

The patient received detailed multimodal imaging investigation and follow-up, but the imaging signs were puzzling. Emergency multiple mode CT images exhibited left MCA hyperdensity sign, low brain tissue density sign covering more than 1/3 of the MCA territory, and low perfusion over the entire MCA territory. Follow-up cranial CT and MR imaging features were consistent with cerebral infarction and reperfusion injury. A TCD study 1 week after onset showed recanalization of the occluded left MCA with high velocity hyperperfusion. Also, repeated MR angiography and perfusion indicated that the left MCA was dilated and the left cerebral hemisphere hyperperfused. Another follow-up TCD at 2 weeks post-onset revealed normal MCA velocity. Considering all imaging features and TCD results, it was still not possible to make a definitive differential diagnosis of cerebral hyperfusion syndrome or reperfusion injury.

The pathogenesis of CHS is not clear,^[2]^ and some case reports^[7–8]^ speculated that damage to pericarotid sympathetic fibers results in loss of vasoconstriction in large and small intracranial arteries, leading to a degree of hypertension exceeding the ameliorative capacity of small arteriole myogenic autoregulation. Alternatively, reperfusion injury has been defined in numerous ways, including activation of endothelium, excess production of oxygen free radicals, inflammatory responses and leukocyte recruitment, increased cytokine production, and edema formation.^[4,9]^ Recent animal studies suggest that CHS and reperfusion injury are distinct pathological entities. In our patient, however, clinical presentation and imaging features were consistent with both. We speculate that infarction resulted in loss of homeostatic vasoconstriction of the MCA and caused hyperperfusion.

## 4 Conclusion

Our patient presented with 2 major neuropathologies—left MCA occlusion of unknown origin followed by spontaneous recanalization, and arterial dilatation and high velocity flow (hyperperfusion) of the recanalized artery. Recanalization promoted brain edema and HT. We speculate that CHS was involved in the reperfusion injury and was also a manifestation of reperfusion. The relationship between CHS and reperfusion injury requires much further study.
